# Wound healing properties of *Gliricidia sepium* leaves from Indonesia and the Philippines in rats (*Rattus norvegicus*)

**DOI:** 10.14202/vetworld.2021.820-824

**Published:** 2021-03-31

**Authors:** Aulaani’am Aulanni’am, Krismal Marchel Ora, Nisa Ain Ariandini, Dyah Kinasih Wuragil, Fajar Shodiq Permata, Wibi Riawan, Ma Asuncion Guiang Beltran

**Affiliations:** 1 Biochemistry Laboratory, Faculty of Sciences, Brawijaya University, Indonesia; 2 Bachelor of Veterinary Student, Faculty of Veterinary Medicine, Brawijaya University, Indonesia; 3 Laboratory of Veterinary Biochemistry, Faculty of Veterinary Medicine, Brawijaya University, Indonesia; 4 Laboratory of Veterinary Histology, Faculty of Veterinary Medicine, Brawijaya University, Indonesia; 5 Department of Molecular and Biochemistry, Faculty of Medicine, Brawijaya University, Indonesia; 6 Department of Microbiology and Veterinary Public Health, College of Veterinary Medicine, Tarlac Agricultural University, The Philippines

**Keywords:** flavonoid, *Gliricidia sepium* leaves, herbal plants, saponin, tannin, wound healing

## Abstract

**Background and Aim:**

*Gliricidia sepium* is a medium-sized leguminous plant found widely in tropical to subtropical areas. It has been used as a medicinal ingredient and in rodenticides by local communities in both Indonesia and the Philippines. This study aimed to investigate the wound healing effects of an ointment containing *G. sepium* leaves on inflammatory cells using a rat model. We also determined its effect on the expression of interleukin (IL) 6 and IL-1β.

**Materials and Methods:**

We used 16 Wistar male rats aged approximately 2 months and weighing 150-200 g. They were divided into four treatment groups (T1, positive control; T2, negative control; T3, wounds treated with *G. sepium* from Indonesia; and T4, wounds treated with *G. sepium* from the Philippines), and the ointment therapies were applied to wounds for 3 days. Hematoxylin and eosin staining was performed to examine the inflammatory cells microscopically. IL-1β and IL-6 expression were observed immunohistochemically.

**Results:**

*G. sepium* leaves significantly (p<0.05) decreased the number of inflammatory cells, and the expression of IL-1β and IL-6 in the group treated with Indonesian *G. sepium* leaves was higher than that in the group treated with *G. sepium* leaves from the Philippines. The leaves contain flavonoids, saponins, and tannins, which act as anti-inflammatory agents to enhance the wound healing process.

**Conclusion:**

Our findings suggest that *G. sepium* leaves from both the Philippines and Indonesia possess wound healing properties.

## Introduction

Injuries occur because of pathological processes caused by internal or external factors that affect specific organs and result in structural and functional damage. Causes of injuries include sharp objects, blunt objects, changes in temperature, chemicals, electric shock, and animal bites [1]. Incision wounds are injuries that occur from cutting with a sharp instrument, such as a scalpel, during surgery. Clean and aseptic wounds are usually closed by sutures [2]. Wound healing occurs when damaged tissue is replaced by new tissue through the processes of regeneration and repair and is divided into four phases: Hemostasis, inflammation, proliferation, and remodeling [3]. Wound healing is a complex process that involves both local and systemic cellular and biochemical responses. The process of tissue repair occurs in the inflammatory phase, where the amount of released inflammatory mediators, such as interleukin (IL) 1β, IL-6, transforming growth factor-β, and tumor necrosis factor-α, is increased [4]. These cytokines act as pro-inflammatory factors that are produced in response to tissue damage, macrophage migration, and the production of other pro-inflammatory cytokines [5].

*Gliricidia sepium* is a leguminous plant that grows quickly in dry areas of Indonesia and the Philippines and is found widely in tropical to subtropical areas [6]. *G. sepiu*m is known as “gamal” in Indonesia and “kakawate” in the Philippines [6]. There have been some studies of its active substances, including flavonoids, saponins, tannins, alkaloids, polyphenols, hydroxyl acid, and coumarin [7]. Some studies have reported that *G. sepiu*m leaves possess anti-inflammatory properties, particularly their flavonoids, which can reduce pain and bleeding, while others have proved their antibacterial and antioxidant properties [8].

We performed this study to further determine the efficacy of *G. sepium* leaves as a wound healing agent based on the evidence of decreased inflammatory cells as well as decreased expression of IL-β and IL-6.

## Materials and Methods

### Ethical approval

The use of animal models in this study was approved by the Brawijaya University Research Ethics Committee (No. 1004-KEP-UB).

### Study period and location

The study was conducted from May to October 2020 at the Animal Disease and Diagnostic Laboratory, Faculty of Veterinary Medicine, Brawijaya University, Malang, Indonesia.

### Animal preparation

We used male Wistar rats (*Rattus norvegicus*) aged approximately 2 months and weighing 150-200 g in our study. The study design was completely randomized, and the rats were divided into four treatment groups comprising four rats per group as follows: T1, positive control, treated with a commercial wound healing agent; T2, negative control; T3, wounds treated with *G. sepium* from Indonesia; and T4, wounds treated with *G. sepium* from the Philippines. The rats were anesthetized with an intramuscular injection of ketamine (10 mg/kg body weight). The back of the rats were shaved and disinfected with 70% alcohol. A 2 cm incision was made subcutaneously in the median portion of the dorsal vertebrae using a scalpel blade. The wound was sewn using silk thread (½ 35 mm) in a simple continuous pattern. Then, the rats were returned to individual cages based on their treatment group.

### *G. sepium* ointment preparation and injury treatment

*G. sepium* leaves from Indonesia and the Philippines were collected and then identified in the Plant Taxonomy Laboratory of the Biology Department at Brawijaya University. The leaves from the Philippines were transported after obtaining an appropriate permit. All leaves were dried, ground into a powder, and made into an ointment by adding a hydrocarbon-based Vaseline ointment base. The ointment was applied to the wounds for 3 days in the designated treatment groups.

### Histopathology preparation and inflammatory cell count

After each group had received the appropriate treatment for 3 days, the rats were sacrificed, and the skin tissue was retrieved. The histopathological examination was conducted based on the previous methods [9], and the inflammatory cells were observed and counted microscopically following staining with hematoxylin and eosin.

### Measurement of IL-1β and IL-6 expression

An immunohistochemistry technique was performed to analyze IL-1β and IL-6 expression based on the previous methods [9]. We used an ImmunoRatio software (available online: http://imtmicroscope.uta.fi/immunoratio/) to observe and analyze the expression of IL-1β and IL-6 by calculating the percentage of the affected area.

### Statistical analysis

Statistical analyses were conducted using SPSS software version 14.0 (IBM, USA). The data were analyzed with one-way analysis of variance (ANOVA) and a Tukey test with α=0.05 to determine differences between the treatment groups.

## Results

### Effect of ointment containing *G. sepium* leaves on inflammatory cells

The macroscopic observation of wound healing in rats differed among the treatment groups. In the positive control group, the wound had not closed and appeared to be in the inflammatory phase, which generally occurs in 2-4 days. In the groups that received ointment containing *G. sepium* leaves from either Indonesia or the Philippines, the incision wound began to close or was completely closed on day 3 (Table-1).

**Table-1:** The number of inflammatory cells in wounds treated with *Gliricidia sepium.*

Groups*	Inflammatory cells expression	Decreasing of inflammatory cells expression (%)
T1 (+)	6.80±3.89^a^	-
T2 (−)	76.32±36.81^b^	69.52
T3 (Indon)	15.40±7.92^a^	79.82
T4 (Phil)	10.20±8.34^a^	86.63

* (T1) positive control, (T2) negative control, (T3) wounds treated with *Gliricidia sepium* from Indonesia, and (T4) wounds treated with *Gliricidia sepium* from the Philippines

### Effect of ointment containing *G. sepium* leaves on IL-1β expression

The expression of IL-1β in the positive control (T1) group was 41.28±9, and this level was used as an indicator of IL-1β expression in normal rats (Table-2).

**Table-2:** The expression of IL-1β.

Groups*	IL-1β expression	Declining of IL-1β (%)
T1 (+)	41.28±9.28%^a^	-
T2 (−)	75.54±11.19%^c^	-
T3 (Indon)	48.68±8.20%^b^	35.55%
T4 (Phil)	28.10±7.35%^a^	62.80%

* (T1) positive control, (T2) negative control, (T3) wounds treated with *Gliricidia sepium* from Indonesia, and (T4) wounds treated with *Gliricidia sepium* from the Philippines. IL=Interleukin

### Effect ointment containing *G. sepium* leaves on IL-6 expression

The results of the one-way ANOVA showed that the administration of ointment containing *G. sepium* leaves from Indonesia (T3) and from the Philippines (T4) significantly reduced the expression of IL-6 (p<0.05) compared with the positive control group (T1) (Table-3).

**Table-3:** The expression of IL-6.

Groups*	IL-6 expression	Declining of IL-1β (%)
T1	24.16±2.12^a^	-
T2	96.86±1.04^d^	-
T3	70.36±1.35^c^	27.35
T4	60.52±2.27^b^	37.58

* (T1) positive control, (T2) negative control, (T3) wounds treated with *Gliricidia sepium* from Indonesia, and (T4) wounds treated with *Gliricidia sepium* from the Philippines. IL=Interleukin

## Discussion

The number of inflammatory cells in the negative control group was the highest and was significantly higher than the positive control and the treatment groups, which indicated that tissue damage had occurred and the inflammatory phase was prolonged compared with the other groups. Macrophages and neutrophils increase tissue damage and increase the phagocytosis of foreign objects. The damaged cells release cytokines as chemotactic factors for inflammatory cells to induce an inflammatory response. Chemotactic factors cause macrophages, lymphocytes, and polymorphonuclear leukocytes (PMNs) to migrate to the wound area [10]. The lowest number of inflammatory cells was observed in the positive control group (T1), which was treated with a commercial wound healing agent. The number of inflammatory cells in the treated groups (T3 and T4) was comparable with T1, meaning *G. sepium* leaves possessed healing properties, as with the commercial preparation. The wounds treated with *G. sepium* leaves from Indonesia showed a decrease in inflammatory cells of 79.82%, while wounds treated with *G. sepium* from the Philippines (T4) showed a decrease of 86.63%. Both therapies showed a significant difference (p<0.05) compared with the positive control (T1).

*G. sepium* leaves contain flavonoids, saponins, tannins, and alkaloids that exert anti-inflammatory properties by inhibiting the activity of the enzymes cyclooxygenase (COX) and lipoxygenase to prevent the release of the histamine during inflammation [11]. Flavonoids also inhibit the accumulation of leukocytes in the inflammatory area, reduce the number of immobilized leukocytes, and inhibit the release of histamine from mast cells. (41.28±9) different cellular mechanism are responsible for anti-inflammation, antimicrobial, and antioxidant which inhibit antioxidant reactions by free radicals and provide nutrients to the skin [12].

**Figure-1:**
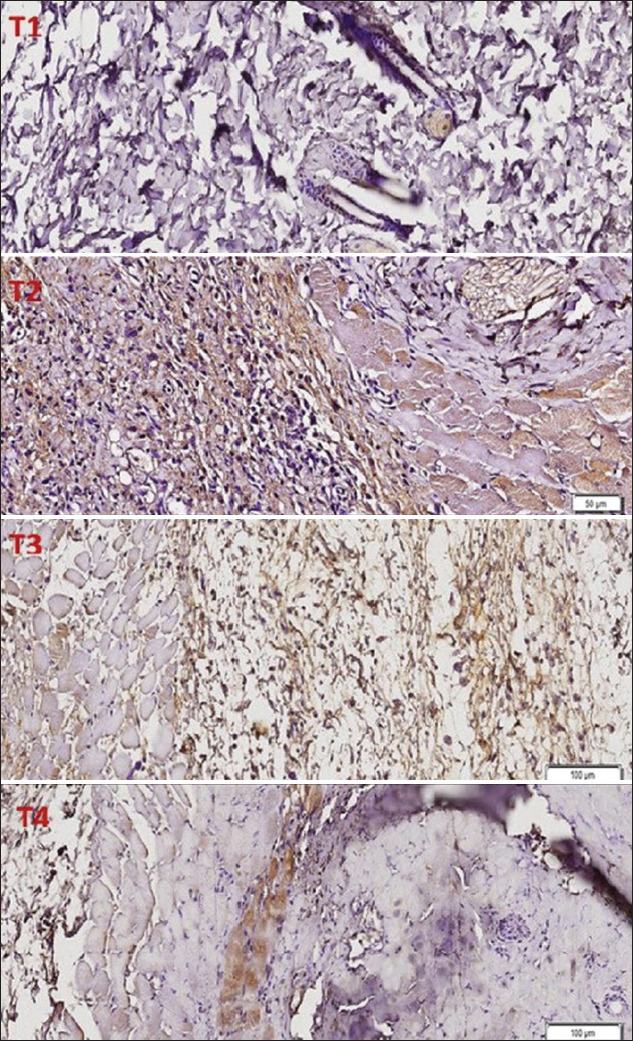
The expression of interleukin-1β on treatment rats.

Under normal skin conditions, the cytokine IL-1β is expressed at low levels in the epidermis of the skin [13]. IL-1β stimulates monocytes and macrophages to produce higher levels of other cytokines that can trigger nuclear factors, such as activators of gene transcription, and trigger an enzyme pathway that turns on prostaglandin activation [14]. IL-1β induces the endothelial excretion of intercellular adhesion molecule 1 and vascular cell adhesion molecule 1 so that inflammatory cells can be identified, which infiltrate the injured area [15]. In the positive control group (T1), the average expression of IL-1β showed a significant difference compared to negative control. Increased expression of IL-1β is observed in inflammation caused by incision wounds. IL-1β activates monocytes and PMNs and can also stimulate inflammation [16]. IL-1β increases the migration of PMNs and monocyte/macrophages to endothelial cells and stimulates the production of prostaglandins and the release of lysosomal enzymes. The continuous production of pro-inflammatory cytokines prolongs the inflammatory phase and the wound healing time [15]. Groups T3 and T4, which were receiving *G. sepium* treatments, showed decreased IL-1β expression of 35.55% and 62.80%, respectively. Furthermore, both treatment groups showed a significant decrease in IL-1β expression when compared with the positive control group (p<0.05). The decrease in IL-1β expression in the T4 group was higher than in the T3 group (Figure-1).

**Figure-2:**
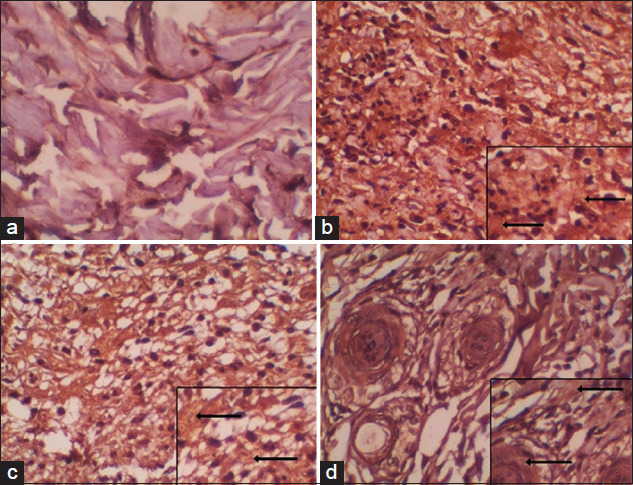
The expression of interleukin-6 on treatment rats: (a) (T1) Positive control, (b) (T2) negative control, (c) (T3) wounds treated with Gliricidia sepium from Indonesia, and (d) (T4) wounds treated with Gliricidia sepium from the Philippines (400×).

*G. sepium* leaves from both Indonesia and the Philippines contain active ingredients, such as flavonoids, saponins, tannins, and alkaloids, that act as antioxidants and anti-inflammatory factors and could inhibit the activity of COX and lipoxygenase and stimulate macrophages to produce growth factors and cytokines to accelerate the wound healing process in the proliferation phase. The results of T1 and T3 were comparable and significantly different from the negative control. *G. sepium* leaves from the Philippines had a better healing effect that was significantly different from *G. sepium* leaves from Indonesia.

The average level of IL-6 expression in the negative control group was 96.86±1.04, and this value was used as an indicator of IL-6 expression in normal rats. Normally, the expression of IL-6 in serum is very high, and it increases in pathological conditions, such as inflammation [17]. The highest IL-6 level was obtained in the negative control group (T2) as the result of the inflammatory response due to injury. IL-6 is a cytokine that causes an acute inflammatory response and plays an essential role in the pathogenesis of inflammatory diseases [18]. It also activates macrophages to produce growth factors needed in the proliferative phase of the wound healing process.

The IL-6 expression levels in the groups treated with *G. sepium* leaves from Indonesia and from the Philippines were significantly different (p<0.05). The highest decrease in IL-6 expression was observed in the positive control (T1) followed by the ointment therapy with *G. sepium* from the Philippines and then the ointment therapy with *G. sepium* from Indonesia. This was thought to be due to the higher saponin and tannin content in *G. sepium* leaves from the Philippines. IL-6 causes macrophages to follow the migration of neutrophils to wounds after 48-72 h, and they become the predominant cells after the 3^rd^ day of injury. Macrophages also play a major role in producing various growth factors required by fibroblasts to produce extracellular matrix in the process of neovascularization. Thus, the presence of macrophages is crucial for wound healing [19]. The flavonoid content is also believed to be beneficial in the wound healing process, and the presence of biosynthetic phase barriers inhibits the production of growth factors and cytokines, such as IL-6, by macrophages, thereby accelerating the phase of proliferation and wound healing [20]. The inflammatory phase begins immediately after the injury until the 5^th^ post-injury day. In inflammatory conditions, various mediators of endothelial derivatives and complement factors attract leukocytes to the endothelial wall. These leukocytes are no longer able to move freely and stimulate neutrophil degranulation. Saponins and tannins can inhibit neutrophil degranulation and reduce the release of arachidonic acid by neutrophils, thereby reducing inflammation [21].

## Conclusion

Our findings have shown that ointment therapy with *G. sepium* leaves from the Philippines to improve wound healing was superior to ointment therapy with *G. sepium* leaves from Indonesia. This was based on decreased levels of inflammatory cells and decreased expression of IL-1β and IL-6 compared with the negative control treatment. Future analysis of the components of *G. sepium* is necessary to prove its efficacy in wound healing.

## Authors’ Contributions

AA, DKW, FSP, and WR designed the research experiments, data analysis, and writing the manuscript for publications. KMO, NAA, and WR conducted the laboratory works as well as results analysis. MAGB conducted data analysis and proofread the manuscript. All authors read and approved the final manuscript.
